# Spin photocurrent spectra induced by Rashba- and Dresselhaus-type circular photogalvanic effect at inter-band excitation in InGaAs/GaAs/AlGaAs step quantum wells

**DOI:** 10.1186/1556-276X-9-130

**Published:** 2014-03-19

**Authors:** Jinling Yu, Shuying Cheng, Yunfeng Lai, Qiao Zheng, Yonghai Chen

**Affiliations:** 1 Institute of Micro/Nano Devices and Solar Cells, School of Physics and Information Engineering, Fuzhou University, Fuzhou 350108, People’s Republic of China; 2 Key Laboratory of Semiconductor Materials Science, Institute of Semiconductors, Chinese Academy of Sciences, P.O. Box 912, Beijing 100083, People’s Republic of China; 3 Key Laboratory of Optoelectronic Materials Chemistry and Physics, Chinese Academy of Sciences, Fuzhou 350002, People’s Republic of China

**Keywords:** Circular photogalvanic effect spectroscopy, Reflectance difference spectroscopy, Rashba and Dresselhaus spin splitting, In-plane optical anisotropy

## Abstract

Spin photocurrent spectra induced by Rashba- and Dresselhaus-type circular photogalvanic effect (CPGE) at inter-band excitation have been experimentally investigated in InGaAs/GaAs/AlGaAs step quantum wells (QWs) at room temperature. The Rashba- and Dresselhaus-induced CPGE spectra are quite similar with each other during the spectral region corresponding to the transition of the excitonic state 1H1E (the first valence subband of heavy hole to the first conduction subband of electrons). The ratio of Rashba- and Dresselhaus-induced CPGE current for the transition 1H1E is estimated to be 8.8±0.1, much larger than that obtained in symmetric QWs (4.95). Compared to symmetric QWs, the reduced well width enhances the Dresselhaus-type spin splitting, but the Rashba-type spin splitting increases more rapidly in the step QWs. Since the degree of the segregation effect of indium atoms and the intensity of build-in field in the step QWs are comparable to those in symmetric QWs, as proved by reflectance difference and photoreflectance spectra, respectively, the larger Rashba-type spin splitting is mainly induced by the additional interface introduced by step structures.

## Background

Recently, spintronics has attracted much attentions due to its significant role in both fundamental research and possible device applications [1-10]. The most critical issues for realizing spintronic devices are the generation and manipulation of spin-polarized carriers in low-dimensional systems [2,11]. Spin-orbit coupling (SOC) and the resulting spin splitting in a two-dimensional system have been used to create and manipulate spin-polarized carriers in nonmagnetic materials without external magnetic field [1,12-14]. There are two kinds of SOC according to different sources of inversion asymmetry: Dresselhaus SOC induced by the bulk inversion asymmetry (BIA), [15] and Rashba SOC induced by structure inversion asymmetry (SIA) [16]. These two terms can interfere with each other and result in an anisotropy of spin splitting. They can cancel each other when the Rashba and Dresselhaus terms have equal strength, which will lead to a zero spin splitting in certain *k* directions. [2] Therefore, it is important to control the value of these two components for spintronic device applications. The Rashba SOC can be tuned by external field [17], uniaxial strain [18,19], and the asymmetric potential gradients in the quantum wells (QWs) [7,8,20], while the Dresselhaus SOC is determined by the materials and the size quantization of the electron wave vector *k* along the growth direction *z*, that is, $\langle k_{z}^{2}\rangle$= (*π*/*w*)^2^ for an infinitely high potential well of width *w*[9]. Nowadays, there are lots of theoretical [21,22] and experimental investigations [7,20] concerning the influence of the asymmetric potential gradients on the spin splitting of the electrons. However, there is seldom report investigating the influence of the asymmetric gradients on the spin splitting when both the electron and holes are involved. Circular photogalvanic effect (CPGE) is an effective experimental tool to measure spin splitting in low-dimensional semiconductor system at room temperature [10], which is induced by unbalanced occupation of carriers in momentum space excited by circularly polarized light as a result of SOC and optical selection rules [4,23]. Spin photocurrent spectra of CPGE excited by inter-band transition, which is firstly observed by Bel’kov et al. [24], are a powerful tool to investigate the spin splitting when both the electron and holes are involved, especially when excitonic effect is dominant [19]. Besides, CPGE current with inter-band resonance excitation shows much stronger intensity than that with inner-band excitation [5]. Thus, some unmeasurable features in the inner-band excitation may be detectable by this highly sensitive inter-band resonance excitation. Step QW structure will not only destroy the structure inversion symmetry by a step potential, but also introduce an additional interface compared to symmetrical QWs. Therefore, step QW structure is of fundamental interest in the study of asymmetric gradient-induced and interface-induced Rashba spin splitting [22].

In this paper, we use CPGE spectra at inter-band excitation to study the Rashba and Dresselhaus spin splitting in an undoped asymmetric In_0.15_Ga_0.85_As/GaAs/AlGaAs step QWs. For an undoped QWs with high crystal quality, the excitonic effect will play a dominant role in the photocurrent spectra. In this case, both of the electron and holes will contribute to the photocurrent [25]. We separate the CPGE spectra induced by Rashba and Dresselhaus spin splitting, respectively, and we find that the Rashba- and Dresselhaus-induced CPGE spectra are quite similar with each other during the spectral region corresponding to the transition of the excitonic state 1H1E (the first valence subband of heavy hole to the first conduction subband of electrons). The ratio of the CPGE current induced by Rashba and Dresselhaus spin splitting for the transition of 1H1E is much larger than that in the symmetric QWs reported in our previous work (i.e., 8.8 vs 4.95). Although the reduced well width enhances the Dresselhaus-type spin splitting compared to the symmetric QWs, the Rashba-type spin splitting in the asymmetry step QWs increases more rapidly. By using reflectance-difference spectrum and photoreflectance spectrum, we find that the degree of the segregation effect of indium atom and the intensity of the build-in field in the step QWs are comparable to those in symmetric QWs. So, the larger Rashba SOC may be mainly induced by the one more interface present in the step structures.

## Methods

The sample studied here is asymmetric In_0.15_Ga_0.85_As/GaAs/Al_0.3_Ga_0.7_As step QWs grown on (001) SI-GaAs substrate by molecular beam epitaxy. After a 2,000-Å buffer layer is grown, ten periods of 50 Å- In_0.15_Ga_0.85_As/50 Å-GaAs/100 Å- Al_0.3_Ga_0.7_As are grown. The grown temperature of In_0.15_Ga_0.85_As and Al_0.3_Ga_0.7_As are 540°C and 580°C, respectively. Then, 500-Å-thick Al_0.3_Ga_0.7_As layer and 100-Å GaAs cap layer are deposited. All epilayers are intentionally undoped and the InGaAs layers are fully strained since their thickness is far below the critical thickness. The sample is cleaved along [110] and [1$\bar{1}$0] (denoted as the *x*^′^ and *y*^′^ directions, respectively) into a square of 5 mm × 5 mm with four pairs of ohmic contacts 4 mm apart along the *x*^′^, *y*^′^ and diagonal directions, respectively, as shown in figure one(a) in [26]. The ohmic contacts are made by indium deposition and annealed at about 420°C in nitrogen atmosphere.

For optical inter-band excitation, a supercontinuum laser source combined with a monochromator is used providing radiation of wavelength in the range between 800 and 950 nm. The supercontinuum laser provides 5-ps pulses with a repetition rate of 40 MHz and an average power of 4 W. Then, the monochromatic light with a linewidth of 1.5 nm goes through a polarizer and a photoelastic modulator (PEM) to yield a periodically oscillating polarization between right (*σ*^-^)- and left (*σ*^+^)-hand circularly polarized light. The light spot on the sample is rectangular of 2 × 3.8 mm with a power of about 150 µW at 950 nm (see figure one(a) in [26]). The photogalvanic current is measured in the unbiased structures at room temperature via a preamplifier and then is recorded by a lock-in amplifier in phase with the PEM. Besides, in order to normalize the data thus enabling a better comparison between BIA and SIA, a common photocurrent *j*_0_ under direct current (dc) bias is also measured by a chopper and a lock-in amplifier. Thus, we can use the common photocurrent *j*_0_ as the denominator for normalizing the CPGE current to eliminate the influences of the anisotropic carrier mobility and carrier density in different directions [26].

For QWs of zinc blende structures grown along the [001] direction, which belongs to *C*_2*v*_ point group symmetry, the Rashba term of the spin-orbital Hamiltonian can be written as [2]

(1) $$H_{R}=\alpha (\sigma_{x}k_{y}-\sigma_{y}k_{x}),$$

while the Dresselhaus term is

(2) $$H_{D}=\beta (\sigma_{x}k_{x}-\sigma_{y}k_{y}).$$

**Figure 1:**
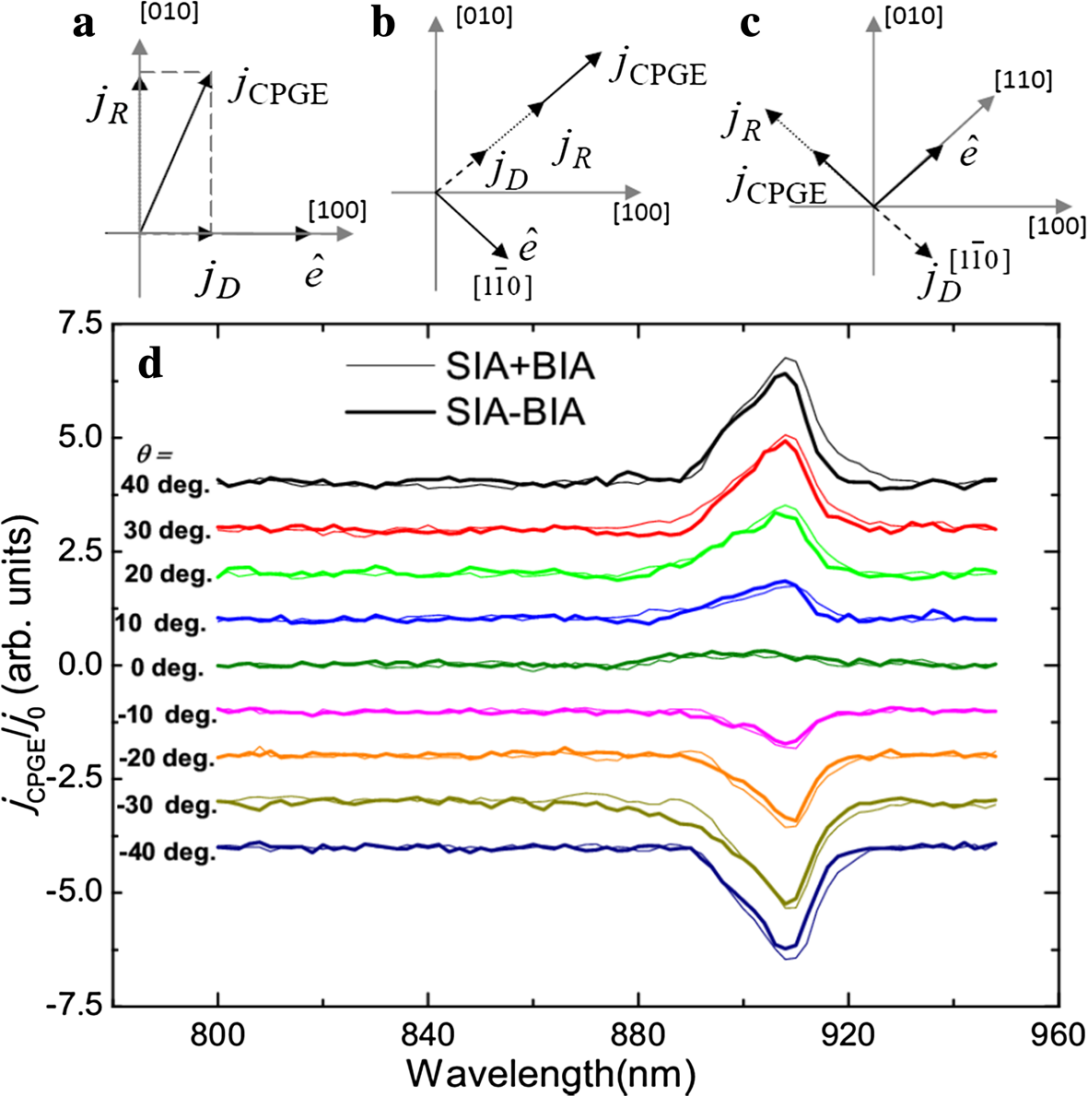
**The schematic diagram of the experimental geometries and the spectra of the normalized CPGE current.** The schematic diagram for geometries CPGE-I **(a)** and CPGE-II **(b and c)**. The spectra of the normalized CPGE current obtained by geometry CPGE-II at different angles of incidence **(d)**. The thin lines indicate the sum of *j*_*R*_ and *j*_*D*_ by the geometry shown in **(b)**, and the thick lines indicate the difference of *j*_*R*_ and *j*_*D*_ obtained by the geometry shown in **(c)**. All of the spectra are shifted vertically for clarity.

Here, *σ* is the Pauli spin matrix, *k* is the in-plane wave vector, *α* (or *β*) is the Rashba (or Dresselhaus) spin-orbital parameter, and the coordinate system is *x*∥ [100] and *y*∥ [010]. These two Hamiltonians will interfere with each other and result in anisotropic spin splitting in *k*-space. We can separate the spin splitting induced by Rashba and Dresselhaus terms according to the method suggested in [4,7], since the Rashba and Dresselhaus terms contribute differently for particular crystallographic directions. Thus, we can use the geometries shown in Figure 1, i.e., named as geometry CPGE-I shown in Figure 1b and geometry CPGE-II shown in Figure 1c,d, to separate the CPGE current induced by Rashba and Dresselhaus SOC, respectively. In the figures,  denotes the direction of light propagation, and *j*_*R*_ and *j*_*D*_ indicate the CPGE current induced by Rashba and Dresselhaus spin splitting, respectively [4,7,26]. Thus, we can obtain *j*_*R*_ and *j*_*D*_ directly from geometry CPGE-I and obtain the sum and difference of *j*_*R*_ and *j*_*D*_ from geometry CPGE-II. Therefore, the *j*_*R*_ and *j*_*D*_ can be obtained separately by the geometry CPGE-I and CPGE-II, respectively, and then be compared to each other to see whether they are self-consistent [26].

In order to get the knowledge of the symmetry of the QW system, we perform reflectance-difference spectrum (RDS) measurement. RDS is an interface-sensitive and nondestructive technique [27,28], and it can precisely measure the in-plane optical anisotropy (IPOA) between the [110] and $\left[1\bar{1}0\right]$ directions. Both of bulk-like and interface-like symmetry reduction effects can introduce IPOA into the (001)-grown zinc blende QWs. The former one can be induced by electric field [29,30], compositional variation across the QWs, uniaxial strain [31,32], and the atomic segregation effect [28], while the latter one can be introduced by anisotropic interface structures [31] and anisotropic interface chemical bonds [33]. Therefore, from the RDS measurement, one can obtain the symmetry properties of QWs. The setup of our RDS is the same as that used in [27], from which we can obtain the relative reflectance difference between [110] and [1$\bar{1}$0] directions, i.e., $\mathrm{\Delta r}/r=2(r_{\left[110\right]}-r_{\left[1\bar{1}0\right]})/(r_{\left[110\right]}+r_{\left[1\bar{1}0\right]})$. Besides, the reflectance spectrum *ΔR*/*R* can be obtained simultaneously during RDS measurements [27,32]. Here, *R* is the reflectivity of the sample, and *ΔR*/*R* is the reflectivity difference of the sample with and without QW layers. To estimate the value of internal field in the sample, we perform PR measurement. The setup of the PR system is the same as that used in [26].

## Results and discussion

**Figure 2:**
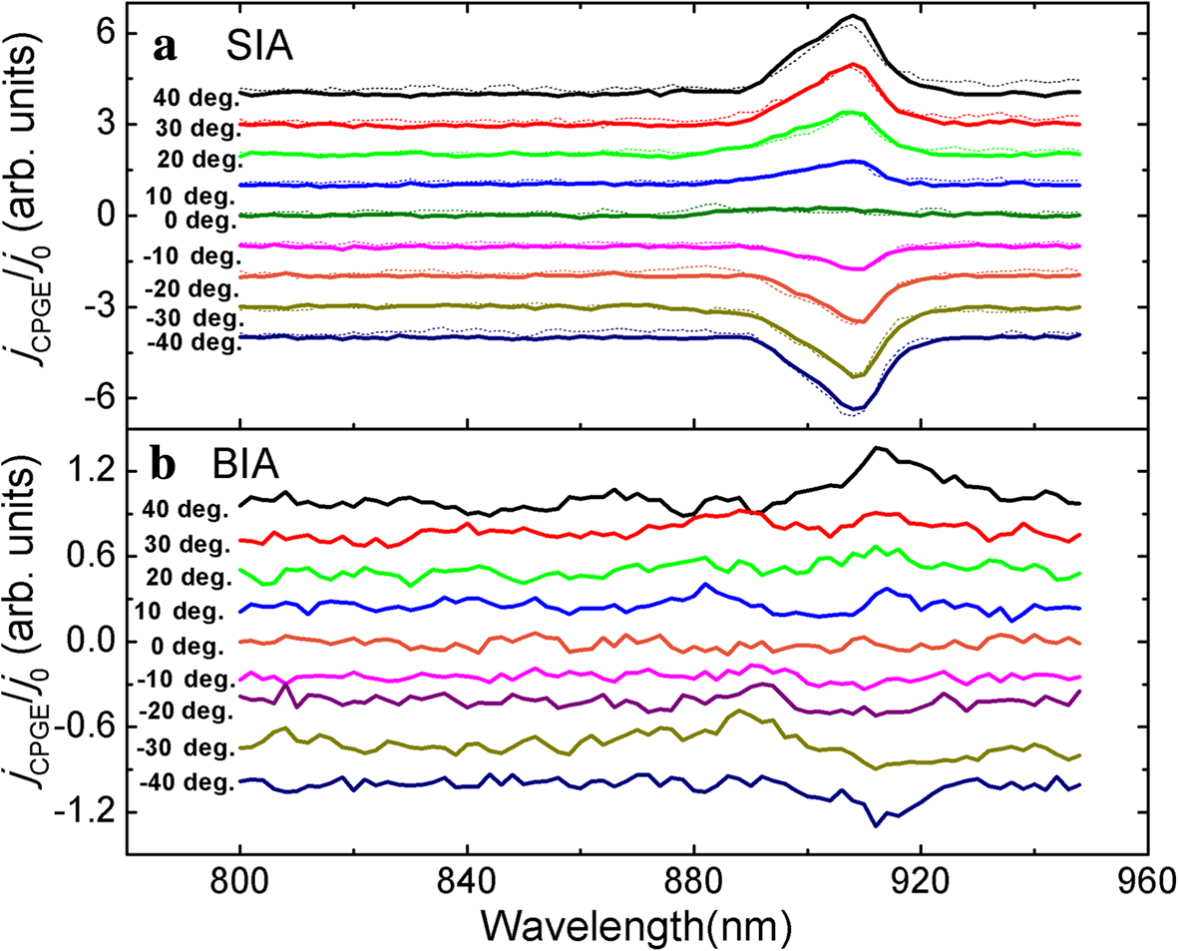
**The normalized SIA- and BIA-induced CPGE current measured at different angles of incidence.(a)** The normalized SIA-induced CPGE current obtained by geometry CPGE-II (thick solid lines) and by geometry CPGE-I (dotted lines). **(b)** The normalized BIA-induced CPGE current obtained by geometry CPGE-II. All of the spectra are shifted vertically for clarity.

**Figure 3:**
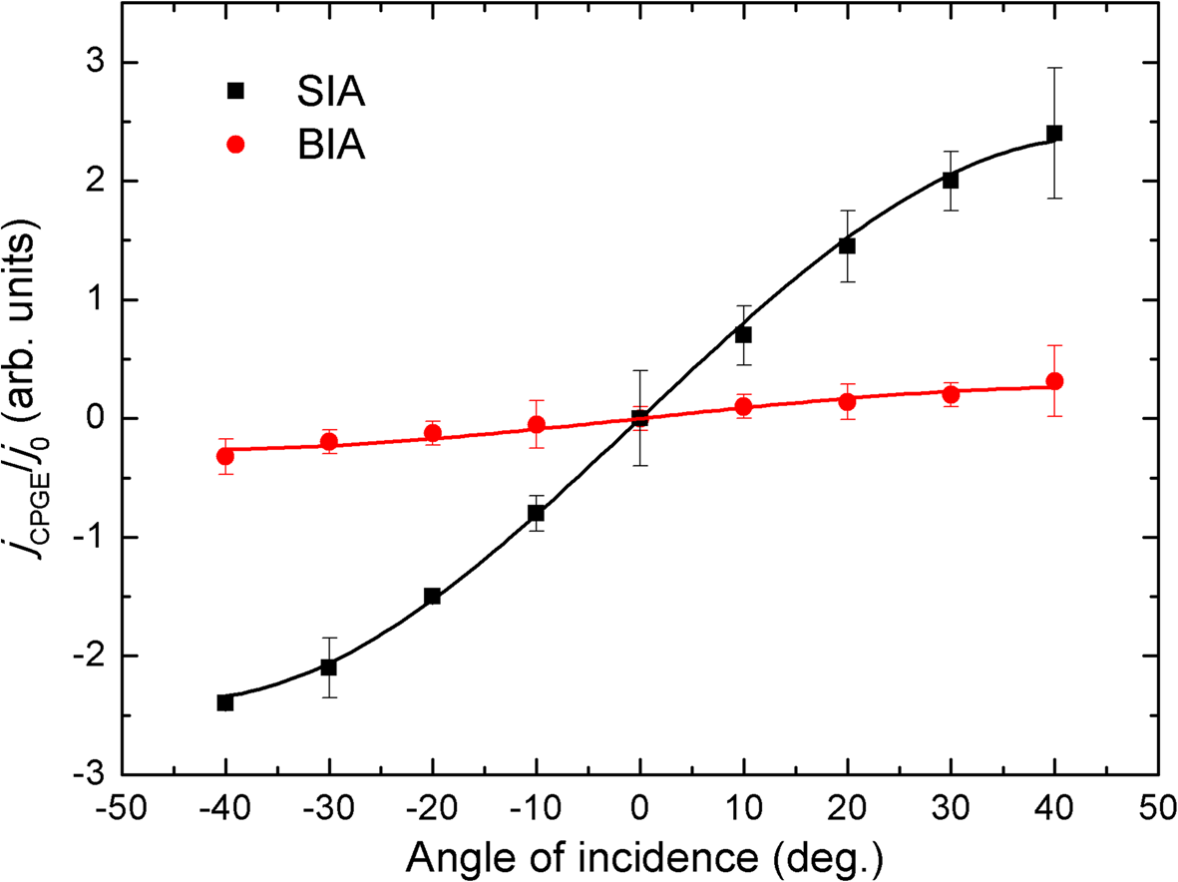
**The magnitude of CPGE current as a function of angle of incidence.** The squares and circles symbols indicate the CPGE current of the excitonic state 1H1E induced by SIA and BIA, respectively. The solid lines are the fitting results.

Figure 1d shows the normalized CPGE current obtained by geometry CPGE-II at different angles of incidence. All of the spectra are shifted vertically for clarity. The thin lines indicate the sum of *j*_*R*_ and *j*_*D*_ obtained by the geometry shown in Figure 1b, and the thick lines are the difference of *j*_*R*_ and *j*_*D*_ obtained by the geometry shown in Figure 1c. It should be noted that the CPGE spectra are only normalized by the common current *j*_0_ at the peak located near 908 nm, which corresponds to the transition of excitonic state 1H1E as discussed below. Thus, we can eliminate the influences of the anisotropic carrier mobility and carrier density in different directions and do not incorporate the spectra dependence signal of *j*_0_ into the CPGE spectra. The power of the exciting light is kept constant during the spectra region between 800 and 950 nm, so it is not necessary to normalize the CPGE spectra by the power of the excitation light. Then, from Figure 1d, we can easily deduce the spectra of the SIA- and BIA-induced CPGE current, which is shown in Figure 2 by thick solid lines. The dotted lines in Figure 2a is the SIA-induced CPGE current obtained by CPGE-I shown in Figure 1a. Unfortunately, the BIA-induced CPGE current is too small to be detected by geometry CPGE-I. From Figure 2a, we can see that the data obtained by the two geometries are consistent with each other. Figure 3 shows the intensity of the CPGE current induced by SIA (squares) and BIA (circles) as a function of angle of incidence corresponding to the transition of the excitonic state 1H1E (at about 908 nm). The solid lines are the fitting results according to the following equation:

(3) $$\begin{array}{c} j_{\text{CPGE}}=\frac{A\sin\theta \cos^{2}\theta }{n(\cos\theta +\sqrt{n^{2}-\sin^{2}\theta })(n^{2}\cos\theta +\sqrt{n^{2}-\sin^{2}\theta })}, \end{array}$$

which describes the dependence of the CPGE current on the angle of incidence *θ* obtained theoretically [2,34]. Here, $A=4E_{0}^{2}\kappa \gamma P_{\text{circ}}$, *E*_0_ is the electric field amplitude of the incident light, *κ* is the absorption coefficient, *γ* = *α* or *β*, *P*_circ_ is the degree of circular polarization, i.e., $P_{\text{circ}}=(I_{\sigma^{+}}-I_{\sigma^{-}})/(I_{\sigma^{+}}+I_{\sigma^{-}})$, and *n* is the refractive index of the QWs material. It can be seen from Figure 3 that the experimental data agree well with the phenomenological theory of CPGE. In the fittings, *n* is adopted to be 3.55 according to [35], and the parameter *A* is fitted to be 1,232 ± 15 and 140 ± 10 for SIA- and BIA-induced CPGE current, respectively. Thus, we can obtain *α*/*β* = 1,232 ± 15 / (140 ± 10) = 8.8 ± 0.1, much larger than the value obtained in symmetric InGaAs/AlGaAs QWs (4.95) investigated in our previous work [26]. This indicates that SIA is the dominant mechanism to induce spin splitting in the step InGaAs/GaAs/AlGaAs QWs. The normalized CPGE signal induced by BIA is estimated to be 0.26 ± 0.01 at an incident angle of 40 °, which is larger than that obtained in the symmetric InGaAs/AlGaAs QWs (0.22 ± 0.01) reported in our previous work [26]. This can be attributed to the size quantization effect of the electron wave vector *k* along the growth direction *z*, since the effective well width is reduced in the step QWs compared to the symmetric QWs, and the Dresselhaus-type spin splitting increases with decreasing well width of QWs according to [9]. Although the Dresselhaus SOC is enhanced in step QWs, the Rashba SOC increases more rapidly, which results in larger RD ratio in the step QWs. In order to find out the reason for the strong Rashba-type spin splitting, we further perform PR and RDS measurements.

**Figure 4:**
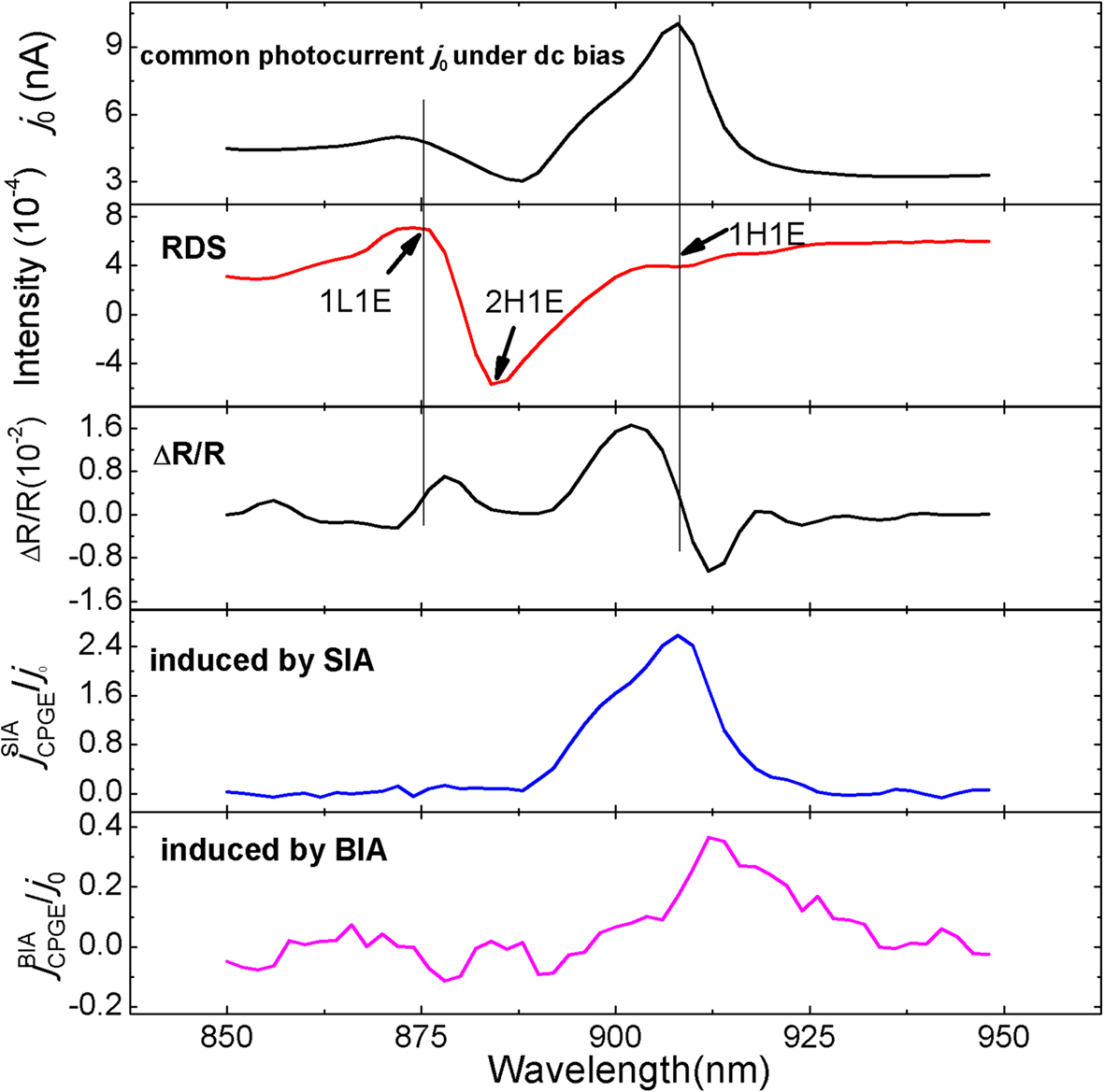
**The comparison of different spectra in the In**_**0.15**_**Ga**_**0.85**_**As/GaAs/Al**_**0.3**_**Ga**_**0.7**_**As step QWs measured at room temperature.** The spectrum of common photocurrent under dc bias (denoted as *j*_0_), the imaginary part of RD spectrum *Δr*/*r*, reflectance spectrum *ΔR*/*R*, and the normalized CPGE current induced by SIA and BIA in the step QWs.

Using the method that has been used in [26], we can estimate the intensity of the internal field to be 12.3 ± 0.4 kV/cm, which is comparable to that in the symmetric QWs (12.6 kV/cm). The imaginary part of RD spectrum *Δr*/*r* is shown in Figure 4, which also shows the spectrum of the common photocurrent under dc bias (denoted as *j*_0_), the reflectance spectrum *ΔR*/*R*, and the spectra of normalized CPGE current induced by SIA and BIA, respectively. By comparing them with each other and performing the theoretical calculation using six-band *k*·*p* theory, we can identify the energy position related to the transitions of the excitonic states 1H1E, 2H1E, and 1L1E, as indicated by the arrows in Figure 4. It can be seen that the peak located near 908 nm in the CPGE spectra is related to the transition of the excitonic state 1H1E in the QWs. From the photoconductivity signal *j*_0_, the 2D density of the photo-induced carriers corresponding to the transition 1H1E is estimated to be about 5 ×10^10^cm^-2^. It also shows that, for the excitonic state 1H1E, the CPGE current induced by SIA has the same sign with that induced by BIA, which is also observed in the symmetric InGaAs/AlGaAs QWs [26] and the GaAs/AlGaAs QWs [19]. This phenomenon is different from that predicted in [3], which shows that the BIA-induced CPGE current is close to zero for the transition of 1H1E. This discrepancy may be attributed to the following two reasons: (1) the prediction is based on the infinitely high-barrier approach, which may introduce some errors; (2) the prediction does not take into account the excitonic effect, which will dominate in the inter-band resonant transition of undoped QWs [19]. There are two ways for the generation of the spin-polarized carriers that form the CPGE current: (1) the direct formation of free electrons and holes, i.e., the direct excitation of electrons from the valence band to the conduction band and (2) the creation of free carriers through excitons [25,36]. Having a neutral charge, excitons themselves cannot contribute to the CPGE current, so they must dissociate in order to make a contribution to the spin photocurrent. There are three mechanisms for the dissociation of excitons to produce free carriers: interaction with (1) phonons, (2) impurity centers, and (3) excitons. The first and the second one are predominant at temperature above and below 70 K, respectively [25,36]. When the excitons make a dominant contribution to the spin photocurrent, the maxima of the photocurrent is always corresponding to the exciton absorption lines. However, for a CPGE current in which the excitons do not play a dominant role, the peak position does not necessarily locate at an energy position which is exactly corresponding to the transition of the excitons [3,5]. Besides, the excitonic-related CPGE current is expected to be much larger than that of the common CPGE, due to its larger absorption coefficient. What is more, the excitonic spin photocurrent is anticipated to show strong temperature dependence effect. Since the excitonic effect is much stronger in low temperature, we expect stronger intensity of the excitonic spin photocurrent in low temperature. The CPGE signal related to the transitions of 2H1E and 1L1E have not been observed in the step QW system, and one of the possible reasons is the weak intensity of the excitation light. It is expected that the CPGE current corresponding to the transition of 1L1E should show the same sign and similar line shape as that of 1H1E, but with lower intensity due to its lower transition probability. The spectra dependence of the CPGE current for the transitions of 1H1E and 1L1E have been observed in the GaAs/AlGaAs QWs [19], and they show the same sign and similar line shape. The CPGE current of the transition of 2H1E is expected to be very weak and difficult to be observed, since it is a forbidden transition with a very low transition probability.

For inter-band excitation of undoped QWs investigated in our case, both electrons and holes may contribute to the CPGE current. Which one plays a dominant role is closely related to their spin relaxation time. The spin relaxation time of electrons in an undoped GaAs/AlGaAs QWs with a well width of 7.5 nm is measured to be 70 ps [37], while that of holes is reported to range from 4 ps [38] to as long as 1,000 ps [39] depending on the doping levels, temperature, and quantum well structures. A recent experiment investigation on p-type QWs concludes that the spin relaxation time of holes should be at least 100 ps and approaching the nanosecond (ns) range at a temperature of 4 K [40]. Besides, a more recent theoretical analysis found that the spin relaxation time can be of the same order of magnitude for electrons and holes for quantum dots with large lateral dimensions [41]. This qualitative conclusion should be of some relevance also for QWs [42]. Therefore, we suppose that the electrons and holes may contribute to the observed CPGE current at the same order.

From the RDS spectrum *Δr*/*r* and the reflectance spectrum *ΔR*/*R*, we can obtain the degree of polarization (DP) for the transitions 1H1E and 1L1E by [26,27]:

(4) DP=|Δr/r|/|ΔR/R|.

**Figure 5:**
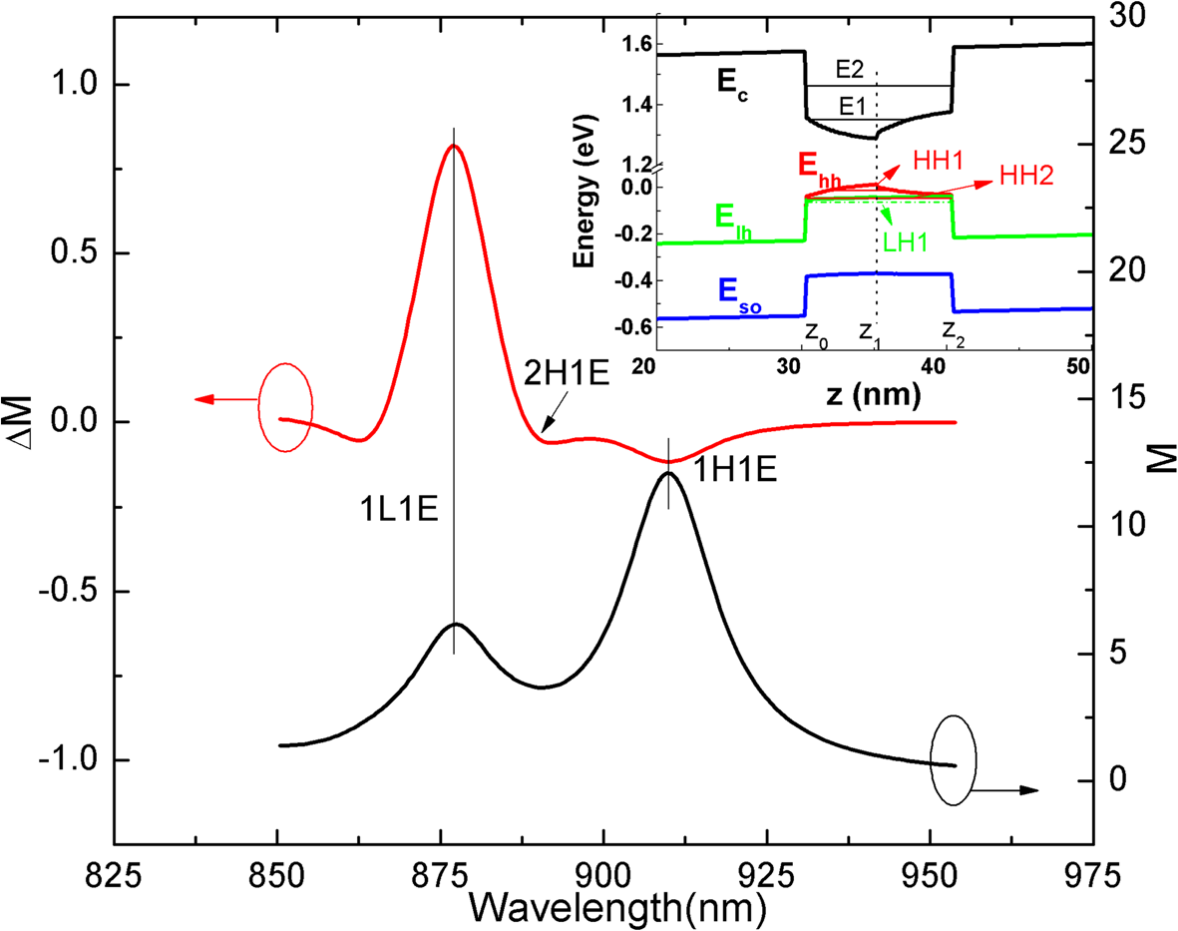
**The calculated anisotropic transition probability*ΔM* and the average transition probability*M*.** The vertical lines and arrows indicate the transition positions of 1H1E, 2H1E, and 1L1E. The inset shows the calculated energy band alignment of In_0.15_Ga_0.85_As/GaAs/Al_0.3_Ga_0.7_As step QWs with segregation length of indium atoms *l* = 2.8 nm and internal field *F* = 12.3 kV/cm. *E*_*c*_, *E*_*lh*_, *E*_*hh*_, and *E*_*so*_ represent the energy band alignment of the electron band, light-hole band, heavy-hole band, and the spin-orbit split-off band, respectively.

Here, DP is defined as $\text{DP}=(M_{\left[110\right]}-M_{\left[1\bar{1}0\right]})/(M_{\left[110\right]}+M_{\left[1\bar{1}0\right]})$, in which *M*_[110]_ is the transition probability when the light is polarized along the [110] direction. In the meantime, we can use *k*·*p* theory, as described in [26], to simulate the DP value theoretically. Specifically speaking, we treat the hole mixing induced by the shear strain *ε*_*xy*_, the electric field, atomic segregation, and anisotropic interface structures as perturbation, and the perturbation Hamiltonian *H*^′^ can be written as [26,33,43,44]

(5) $$\begin{array}{c} H^{\prime }=\left(\begin{array}{cccccc} 0 & 0 & \text{iR} & 0 & 0 & \text{iR}\\ 0 & 0 & 0 & \text{iR} & Q & 0\\ -iR^{†} & 0 & 0 & 0 & 0 & Q\\ 0 & -iR^{†} & 0 & 0 & -iR^{†} & 0\\ 0 & Q & 0 & \text{iR} & 0 & 0\\ -iR^{†} & 0 & Q & 0 & 0 & 0 \end{array}\right) \end{array}$$

with [27,31]

(6) $$\begin{array}{cc} R(z)= & \left\{{\text{Dd}}_{14}\;F+D\epsilon_{\text{xy}}+\left[\frac{x_{1}P_{1}}{l_{1}}\exp(-\frac{z-z_{0}}{l_{1}})\Theta (z-z_{0})\right)\right)\\ & -\frac{x_{1}P_{2}}{l_{2}}\exp(-\frac{z-z_{1}}{l_{2}})\Theta (z-z_{1})\\ & \left(\left(-\frac{x_{2}P_{3}}{l_{3}}\exp(-\frac{z-z_{2}}{l_{3}})\Theta (z-z_{2})\right]\right\}, \end{array}$$

and [43]

(7) $$Q=-\frac{b}{2}(\epsilon_{\text{xx}}+\epsilon_{\text{yy}}-2\epsilon_{\text{zz}}),$$

for the basis |3/2,3/2 >,|3/2,1/2 >,|3/2,-1/2 >,|3/2,-3/2 >,|1/2,1/2 >, and |1/2,-1/2 >. Here *b* and *D* are the Bir-Pikus deformation potentials, *F* is the electric field along the [001] direction, *d*_14_ is the piezoelectric constant, *ε*_*ij*_ denotes the symmetric strain tensor, *z* = *z*_0_ (*z*_1_ or *z*_2_) is the location of the interfaces of QWs (see the inset in Figure 5), *P*_1_ (*P*_2_ or *P*_3_) is the interface potential parameter describing the effect of *C*_2*v*_ interface symmetry at interface located at *z*_0_ (*z*_1_ or *z*_2_) [27], *x*_1_ and *x*_2_ are the concentrations of In and Al, respectively, with the assumption that the value of the interface potential is proportional to the components of In or Al elements at interface [27], and *l*_1_ (*l*_2_ or *l*_3_) is the segregation length of the indium atoms in interface located at *z*_0_ (*z*_1_ or *z*_2_). The segregation model developed by Muraki [45] is adopted, which assumes that the segregation lengths of the indium atoms on the interfaces to be equal. The energies and wave functions of the holes and electrons are calculated by six-band and two-band *k*·*p* theory, respectively. Then, the anisotropic transition spectrum $\mathrm{\Delta M}=M_{\left[110\right]}-M_{\left[1\bar{1}0\right]}$ and the averaged transition spectrum *M* ($M=(M_{\left[110\right]}+M_{\left[1\bar{1}0\right]})/2$) are simulated using the following equation [26]:

(8) $$\begin{array}{ll} M(\text{or}\mathrm{\Delta M})= & \underset{n,m}{\sum }\frac{1}{\pi }\frac{0.5\Gamma }{{\left(E-E_{\text{nm}}\right)}^{2}+{\left(0.5\Gamma \right)}^{2}}\\ & \times P_{\text{nm}}(\text{or}\Delta P_{\text{nm}}). \end{array}$$

Here, *Γ* is the linewidth of the transition, and *E*_*nm*_(*P*_*nm*_) is the energy (probability) of the transition between *n*E (the *n*th conduction subband of electrons) and *m*LH (the *m*th valence subband of light holes) or between *n*E and *m*HH. Thus, by fitting the theoretical calculated DP with that obtained by experiments, we can determine the structure parameters of the QWs, such as the interface potential parameters *P*_*i*_ (*i* = 1, 2, 3), segregation length of atoms *l*_*i*_ (*i* = 1, 2, 3), and anisotropy strain *ε*_*xy*_.

Using Equation 4, we can estimate the DP values of the transition for the excitonic states 1H1E and 1L1E to be 0.5 *%* ± 0.5*%* and 6.3 *%* ± 0.5*%*, respectively. In order to calculate the theoretical DP value of the transitions of the QWs, we should first estimate the interface potential *P*_0_ for an ideal InAs-Al_0.3_Ga_0.7_As, GaAs-InAs, and AlAs-GaAs interfaces, respectively. Using the perturbed interface potential, the averaged hybrid energy difference of interface, and the lattice mismatch models, and then adding them up, we can obtain the value of *P*_0_ for an ideal InAs-Al_0.3_Ga_0.7_As interface to be 639 meV Å [46]. The *P*_0_ at GaAs-InAs and AlAs-GaAs interfaces are reported to be 595 and 400 meV Å [27,47], respectively. Since the InAs-on-Al_0.3_Ga_0.7_As interface tends to be an ideal and abrupt interface, we adopt *P*_1_ = *P*_0_. Due to the segregation effect of indium atoms at the GaAs-on-InAs interface, *P*_2_ may not be equal to *P*_0_. Therefore, we treat *P*_2_ as a fitting parameter. According to [27], the interface potential *P*_3_ for AlAs-on-GaAs interface is fitted to be 440 meV Å, due to the anisotropic interface structures. Thus, adopting *P*_1_ = 639 meV Å, *P*_3_ = 440 meV Å, and internal electric field *F* = 12.3 kV/cm (obtained by PR measurements) and treating the interface potential *P*_2_ and the segregation length *l*_1_ = *l*_2_ = *l*_3_ = *l* as fitting parameters, we fit the theoretical calculated DP value to that of experiments. When we adopt *P*_2_ = 650 meV Å, *l* = 2.8 nm, the DP values of the transition 1H1E and 1L1E can be well fitted, and the main features of the RD spectrum are all well simulated (see Figure 5, *ΔM*∝*Δr*/*r*). The large segregation effect of indium atoms and the strong internal field reduce the step well into an irregular well, as shown in the inset of Figure 5. This will result in large Rashba spin splitting according to [8,26]. However, we find that the intensity of the internal field and the segregation length of the indium atoms for the step QWs are comparable to those in symmetric QWs, which indicate that the Rashba SOC induced by these two factors are at the same scale and they are not the main reasons for the larger Rashba spin splitting in the step QWs. On the other hand, the interface in QWs will also introduce Rashba-type spin splitting, which is related to some band discontinuities in valence bands at hetero-interfaces [22,48]. Since the step QW structures will introduce one additional interface compared to symmetric QWs and this additional interface will introduce additional Rashba spin splitting, the larger Rashba spin splitting in the step QWs may be mainly induced by this interface Rashba effect. It is worth mentioning that the interface or the segregation effect alone will not necessarily lead to larger Rashba spin splitting, and only when they are combined with large electric field or the presence of a Hartree potential gradient in the asymmetric system will finally result in a significant spin splitting [48].

## Conclusions

In conclusion, we have experimentally investigated the spin photocurrent spectra induced by Rashba- and Dresselhaus-type CPGE at inter-band excitation in InGaAs/GaAs/AlGaAs step QWs at room temperature. It is found that the line shape of CPGE spectrum induced by Rashba SOC is quite similar to that induced by Dresselhaus SOC during the spectral region corresponding to the transition of the excitonic state 1H1E. The ratio of Rashba- and Dresselhaus-induced CPGE current for the transition of the excitonic state 1H1E is estimated to be 8.8 ± 0.1, much larger than that reported in the symmetric QWs in our previous work (i.e., 4.95 in [26]). We also find that, compared to symmetric QWs, the reduced well width in the step QWs enhances the Dresselhaus-type spin splitting, while the Rashba-type spin splitting increases more rapidly. Since the intensity of the build-in field and the degree of the segregation effect in the step QWs are comparable to those in symmetric QWs, which are evident from RDS and PR measurements, the larger Rashba spin splitting in the step QWs are mainly induced by the additional interface introduced by step structures.

## Abbreviations

BIA: bulk inversion asymmetry; CPGE: circular photogalvanic effect; DP: degree of polarization; IPOA: in-plane optical anisotropy; PEM: photoelastic modulator; PR: photoreflectance; QWs: quantum wells; RDS: reflectance-difference spectrum; SIA: structure inversion asymmetry; SOC: spin-orbit coupling; 1H1E: the first valence subband of heavy hole to the first conduction subband of electrons.

## Competing interests

The authors declare that they have no competing interests.

## Authors’ contributions

JY conducted the experiments and wrote the paper. YC designed the experiments and performed the sample fabrications. SC, YL, and QZ assisted with the measurements and analysis. All authors contributed through scientific discussions and read and approved the final manuscript.

## References

[B1] Wunderlich J Irvine AC Sinova J Park BG Zarbo LP Xu XL Kaestner B Novak V Jungwirth T **Spin-injection hall effect in a planar photovoltaic cell** Nat Phys 2009 9 9 675 681 10.1038/nphys1359

[B2] Ganichev SD Prettl W **Spin photocurrents in quantum wells** J Phys-Condensed Matt 2003 9 20 935 983 10.1088/0953-8984/15/20/204

[B3] Golub LE **Spin-splitting-induced photogalvanic effect in quantum wells** Physical Review B 2003 9 23 235320

[B4] Ganichev SD Bel’kov VV Golub LE Ivchenko EL Schneider P Giglberger S Eroms J De Boeck J Borghs G Wegscheider W Weiss D Prettl W **Experimental separation of Rashba and Dresselhaus spin splittings in semiconductor quantum wells** Phys Rev Lett 2004 9 25 256601 15245041 10.1103/PhysRevLett.92.256601

[B5] Yang CL He HT Ding L Cui LJ Zeng YP Wang JN Ge WK **Spectral dependence of spin photocurrent and current-induced spin polarization in an InGaAs/InAlAs two-dimensional electron gas** Phys Rev Lett 2006 9 18 186605 16712387 10.1103/PhysRevLett.96.186605

[B6] Cho KS Liang CT Chen YF Tang YQ Shen B **Spin-dependent photocurrent induced by Rashba-type spin splitting in Al**_ ** *0.25* ** _**Ga**_ ** *0.75* ** _**N/GaN heterostructures** Phys Rev B 2007 9 8 085327

[B7] Giglberger S Golub LE Bel’kov VV Danilov SN Schuh D Gerl C Rohlfing F Stahl J Wegscheider W Weiss D Prettl W Ganichev SD **Rashba and Dresselhaus spin splittings in semiconductor quantum wells measured by spin photocurrents** Phys Rev B 2007 9 3 035327 10.1103/PhysRevLett.92.256601 15245041

[B8] Eldridge PS Leyland WJH Lagoudakis PG Harley RT Phillips RT Winkler R Henini M Taylor D **Rashba spin-splitting of electrons in asymmetric quantum wells** Phys Rev B 2010 9 4 045317

[B9] Walser MP Siegenthaler U Lechner V Schuh D Ganichev SD Wegscheider W Salis G **Dependence of the Dresselhaus spin-orbit interaction on the quantum well width** Phys Rev B 2012 9 19 195309

[B10] Yin C Yuan H Wang X Liu S Zhang S Tang N Xu F Chen Z Shimotani H Iwasa Y Chen Y Ge W Shen B **Tunable surface electron spin splitting with electric double-layer transistors based on InN** Nano Lett 2013 9 5 2024 2029 10.1021/nl400153p 23614498

[B11] Awschalom DD Flatte ME **Challenges for semiconductor spintronics** Nat Phys 2007 9 3 153 159 10.1038/nphys551

[B12] Wunderlich J Park BG Irvine AC Zarbo LP Rozkotova E Nemec P Novak V Sinova J Jungwirth T **Spin hall effect transistor** Science 2010 9 6012 1801 1804 10.1126/science.1195816 21205664

[B13] Fiederling R Keim M Reuscher G Ossau W Schmidt G Waag A Molenkamp LW **Injection and detection of a spin-polarized current in a light-emitting diode** Nature 1999 9 6763 787 790 10.1038/45502

[B14] Kotissek P Bailleul M Sperl M Spitzer A Schuh D Wegscheider W Back CH Bayreuther G **Cross-sectional imaging of spin injection into a semiconductor** Nat Phys 2007 9 12 872 877 10.1038/nphys734

[B15] Dresselhaus G **Spin-orbit coupling effects in zinc blende structures** Phys Rev 1955 9 2 580 586 10.1103/PhysRev.100.580

[B16] Bychkov YA Rashba EI **Oscillatory effects and the magnetic susceptibility of carriers in inversion layers** J Phys C Solid State Phys 1984 9 6039 10.1088/0022-3719/17/33/015

[B17] Nitta J Akazaki T Takayanagi H Enoki T **Gate control of spin-orbit interaction in an inverted In(0.53)Ga(0.47)As/In(0.52)Al(0.48)As heterostructure** Phys Rev Lett 1997 9 7 1335 1338 10.1103/PhysRevLett.78.1335

[B18] He XW Shen B Tang YQ Tang N Yin C. M Xu FJ Yang Z. J Zhang GY Chen YH Tang CG Wang ZG **Circular photogalvanic effect of the two-dimensional electron gas in Al**_ ** *x* ** _**Ga**_ ** *1-x* ** _**N/GaN heterostructures under uniaxial strain** Appl Phys Lett 2007 9 7 071912 10.1063/1.2768918

[B19] Yu JL Chen YH Jiang CY Liu Y Ma H Zhu LP **Spectra of Rashba- and Dresselhaus-type circular photogalvanic effect at inter-band excitation in GaAs/AlGaAs quantum wells and their behaviors under external strain** Appl Phys Lett 2012 9 152110 10.1063/1.3702826

[B20] Averkiev NS Golub LE Gurevich AS Evtikhiev VP Kochereshko VP Platonov AV Shkolnik AS Efimov YP **Spin-relaxation anisotropy in asymmetrical (001) Al**_ ** *x* ** _**Ga**_ ** *1-x* ** _**As quantum wells from Hanle-effect measurements: relative strengths of Rashba and Dresselhaus spin-orbit coupling** Phys Rev B 2006 9 033305

[B21] de Andrada e Silva EA La Rocca GC Bassani F **Spin-orbit splitting of electronic states in semiconductor asymmetric quantum wells** Physical Review B 1997 9 16293 16299 10.1103/PhysRevB.55.16293

[B22] Hao YF Chen YH Liu Y Wang ZG **Spin splitting of conduction subbands in Al**_ ** *0.3* ** _**Ga**_ ** *0.7* ** _**As/GaAs/Al**_ ** *x* ** _** *Ga* **_ ** *1-x* ** _**As/Al**_ ** *0.3* ** _**Ga**_ ** *0.7* ** _**As step quantum wells** Europhys Lett 2009 9 37003 10.1209/0295-5075/85/37003

[B23] Cho KS Chen YF Tang YQ Shen B **Photogalvanic effects for interband absorption in AlGaN/GaN superlattices** Appl Phys Lett 2007 9 4 041909 10.1063/1.2435591

[B24] Bel’kov VV Ganichev SD Schneider P Back C Oestreich M Rudolph J Hagele D Golub LE Wegscheider W Prettl W **Circular photogalvanic effect at inter-band excitation in semiconductor quantum wells** Solid State Commun 2003 9 8 283 286 10.1016/j.ssc.2003.08.022

[B25] Yu JL Chen YH Jiang CY Liu Y Ma H Zhu LP **Observation of the photoinduced anomalous hall effect spectra in insulating InGaAs/AlGaAs quantum wells at room temperature** Appl Phys Lett 2012 9 142109 10.1063/1.3701281

[B26] Yu JL Chen Y. H Jiang CY Liu Y Ma H **Room-temperature spin photocurrent spectra at interband excitation and comparison with reflectance-difference spectroscopy in InGaAs/AlGaAs quantum wells** J Appl Phys 2011 9 5 053519 10.1063/1.3555099

[B27] Chen YH Ye XL Wang JZ Wang ZG Yang Z **Interface-related in-plane optical anisotropy in GaAs/Al**_ ** *x* ** _**Ga**_ ** *1-x* ** _**As single-quantum-well structures studied by reflectance difference spectroscopy** Phys Rev B 2002 9 19 195321

[B28] Ye XL Chen YH Xu B Wang ZG **Detection of indium segregation effects in InGaAs/GaAs quantum wells using reflectance-difference spectrometry** Materials Science and Engineering B-Solid State Materials for Advanced Technol 2002 9 62 65

[B29] Zhu BF Chang YC **Inversion asymmetry, hole mixing, and enhanced Pockels effect in quantum wells and superlattices** Phys Rev B 1994 9 11932 10.1103/PhysRevB.50.11932 9975334

[B30] Kwok SH Grahn HT Ploog K Merlin R **Giant electropleochroism in GaAs-(Al,Ga) as heterostructures - the quantum-well Pockels effect** Phys Rev Lett 1992 9 6 973 976 10.1103/PhysRevLett.69.973 10047082

[B31] Tang CG Chen YH Xu B Ye XL Wang ZG **Well-width dependence of in-plane optical anisotropy in (001) GaAs/AlGaAs quantum wells induced by in-plane uniaxial strain and interface asymmetry** J Appl Phys 2009 9 10 103108 10.1063/1.3132089

[B32] Tang CG Chen YH Ye XL Wang ZG Zhang WF **Strain-induced in-plane optical anisotropy in (001) GaAs/AlGaAs superlattice studied by reflectance difference spectroscopy** J Appl Phys 2006 9 11 113122 10.1063/1.2399308

[B33] Krebs O Voisin P **Giant optical anisotropy of semiconductor heterostructures with no common atom and the quantum-confined Pockels effect** Phys Rev Lett 1996 9 1829 10.1103/PhysRevLett.77.1829 10063182

[B34] Yu J Chen Y Cheng S Lai Y **Spectra of circular and linear photogalvanic effect at inter-band excitation in In**_ ** *0.15* ** _**Ga**_ ** *0.85* ** _**As/Al**_ ** *0.3* ** _**Ga**_ ** *0.7* ** _**As multiple quantum wells** Phys E: Low-dimensional Systems and Nanostructures 2013 9 0 92 96

[B35] Takagi T **Refractive index of Ga**_ ** *1-x* ** _**In**_ ** *x* ** _**As prepared by vapor-phase epitaxy** Japanese J Appl Phys 1978 9 1813 1817 10.1143/JJAP.17.1813

[B36] Park YS Reynolds DSC **Exciton structure in photoconductivity of CdS, CdSe, and CdS: Se single crystals** Phys Rev 1963 9 2450 2457 10.1103/PhysRev.132.2450

[B37] Ohno Y Terauchi R Adachi T Matsukura F Ohno H **Spin relaxation in GaAs(110) quantum wells** Phys Rev Lett 9 4196 4199

[B38] Damen TC Via L Cunningham JE Shah J Sham LJ **Subpicosecond spin relaxation dynamics of excitons and free carriers in GaAs quantum wells** Phys Rev Lett 1991 9 3432 3435 10.1103/PhysRevLett.67.3432 10044732

[B39] Roussignol P Rolland P Ferreira R Delalande C Bastard G Vinattieri A Martinez-Pastor J Carraresi L Colocci M Palmier JF Etienne B **Hole polarization and slow hole-spin relaxation in an n-doped quantum-well structure** Phys Rev B 1992 9 7292 7295 10.1103/physrevb.46.7292 10002456

[B40] Mattana R George J-M Jaffrès H Nguyen Van Dau F Fert A Lépine B Guivarc’h A Jézéquel G **Electrical detection of spin accumulation in a p-type GaAs quantum well** Phys Rev Lett 2003 9 166601 12731988 10.1103/PhysRevLett.90.166601

[B41] Bulaev DV Loss D **Spin relaxation and decoherence of holes in quantum dots** Phys Rev Lett 2005 9 076805 16196813 10.1103/PhysRevLett.95.076805

[B42] Gvozdic DM Ekenberg U **Superefficient electric-field-induced spin-orbit splitting in strained p-type quantum wells** Europhys Lett 2006 9 927 10.1209/epl/i2005-10482-6

[B43] Chao CY Chuang SL **Spin-orbit-coupling effects on the valence-band structure of strained semiconductor quantum wells** Physical Review B 1992 9 7 4110 10.1103/physrevb.46.4110 10004141

[B44] Foreman BA **Analytical envelope-function theory of interface band mixing** Phys Rev Lett 1998 9 2 425 10.1103/PhysRevLett.81.425

[B45] Muraki K Fukatsu S Shiraki Y Ito R **Surface segregation of in atoms during molecular-beam epitaxy and its influence on the energy-levels in InGaAs/GaAs quantum-wells** Appl Phys Lett 1992 9 5 557 559 10.1063/1.107835

[B46] Chen YH Wang ZG Yang ZY **A new interface anisotropic potential of zinc-blende semiconductor interface induced by lattice mismatch** Chinese Phys Lett 1999 9 1 56 58 10.1088/0256-307X/16/1/020

[B47] Yu JL Chen YH Tang CG Jiang CY Ye X **Observation of strong anisotropic forbidden transitions in (001) InGaAs/GaAs single-quantum well by reflectance-difference spectroscopy and its behavior under uniaxial strain** Nanoscale Research Letters 2011 9 210 10.1186/1556-276X-6-210 21711728 PMC3211267

[B48] Lin Y Koga T Nitta J **Effect of an InP/In**_ ** *0.53* ** _**Ga**_ ** *0.47* ** _**As interface on spin-orbit interaction in In**_ ** *0.52* ** _**Al**_ ** *0.48* ** _**As/In**_ ** *0.53* ** _**Ga**_ ** *0.47* ** _**As heterostructures** Phys Rev B 2005 9 045328

